# Quadrigeminal cistern arachnoid cyst causing hydrocephalus

**DOI:** 10.11604/pamj.2020.35.27.19768

**Published:** 2020-02-04

**Authors:** Sidi Salem-Memou, Najat Boukhrissi

**Affiliations:** 1 Department of Neurosurgery, National Hospital of Nouakchott, Mauritania

**Keywords:** Arachnoid cyst, neuroendoscopy, quadrigeminal cistern

## Image in medicine

The quadrigeminal cistern is an unusual location for these cysts. Quadrigeminal arachnoid cysts account for 5% to 10% of all intracranial arachnoid cysts. They are frequently diagnosed coincidentally on computed tomography (CT) or magnetic resonance imaging (MRI). They cause symptoms when they become sufficiently large to compress the adjacent brain structures. Endoscopic fenestration of the cyst with cystocisternostomy or cysto ventriculostomy, when combined with third ventriculostomy, is the procedure of choice for such patients. A 14-month-old boywas referred to neurosurgery clinic due to increased head circumference. His had been in good health until age 8 months, between age 9 and 13 months his acquisition of developmental skills was slow. Magnetic resonance image (MRI) demonstrated enlargement of the supratentorial ventricular system secondary to a large quadrigeminal cistern arachnoid cyst compressing the brainstem, cerebellum, aqueduct of Sylvius and fourth ventricle. The cystic mass appeared hyperintense on T2 weighted-images (A, B), hypointense on T1-WI (C, D), similar to cerebrospinal fluid (CSF) signal, without enhancement after Gadolinium administration. The patient was operated by transventricular approach with endoscopic third ventriculocystostomy and endoscopic third ventriculostomy (ETV). The postoperative course was uneventful.

**Figure 1:**
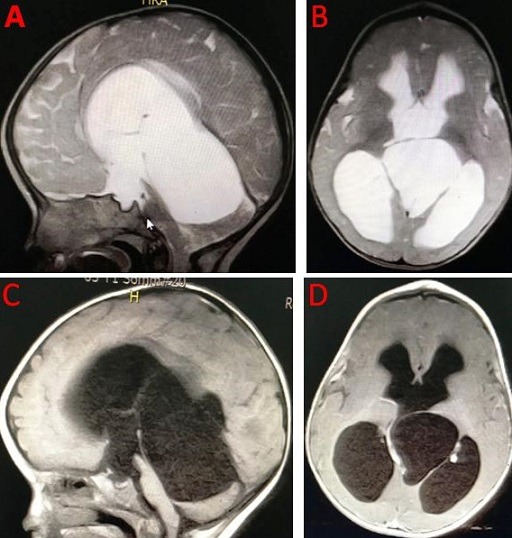
Magnetic resonance image (MRI) demonstrated enlargement of the supratentorial ventricular system secondary to a large quadrigeminal cistern arachnoid cyst compressing the brainstem, cerebellum, aqueduct of sylvius and fourth ventricle. The cystic mass appeared hyperintense on T2 weighted-images (A, B), hypointense on T1-WI (C, D), similar to cerebrospinal fluid (CSF) signal, without enhancement after Gadolinium administration

